# No Evidence of Coronaviruses or Other Potentially Zoonotic Viruses in Sunda pangolins (*Manis javanica*) Entering the Wildlife Trade via Malaysia

**DOI:** 10.1007/s10393-020-01503-x

**Published:** 2020-11-23

**Authors:** Jimmy Lee, Tom Hughes, Mei-Ho Lee, Hume Field, Jeffrine Japning Rovie-Ryan, Frankie Thomas Sitam, Symphorosa Sipangkui, Senthilvel K. S. S. Nathan, Diana Ramirez, Subbiah Vijay Kumar, Helen Lasimbang, Jonathan H. Epstein, Peter Daszak

**Affiliations:** 1 EcoHealth Alliance, 520 Eighth Avenue, Suite 1200, New York, NY 10018 USA; 2 Conservation Medicine, Unit 13H Villamas, Jalan Villamas, 47000 Sungai Buloh, Selangor Malaysia; 3 National Wildlife Forensic Laboratory, Department of Wildlife and National Parks (PERHILITAN), Peninsular Malaysia, KM 10, Jalan Cheras, 56100 Kuala Lumpur, Malaysia; 4 Sabah Wildlife Department, 5th Floor, B Block, Wisma MUIS, 88100 Kota Kinabalu, Sabah, Malaysia; 5 Biotechnology Research Institute, Universiti Malaysia Sabah, Jalan UMS, 88400 Kota Kinabalu, Sabah Malaysia; 6 Faculty of Medicine and Health Sciences, Universiti Malaysia Sabah, Jalan UMS, 88400 Kota Kinabalu, Sabah Malaysia

**Keywords:** Sunda pangolins, SARSr-CoV, Malaysia, COVID-19, Zoonotic viruses, Coronavirus, Wildlife trade

## Abstract

The legal and illegal trade in wildlife for food, medicine and other products is a globally significant threat to biodiversity that is also responsible for the emergence of pathogens that threaten human and livestock health and our global economy. Trade in wildlife likely played a role in the origin of COVID-19, and viruses closely related to SARS-CoV-2 have been identified in bats and pangolins, both traded widely. To investigate the possible role of pangolins as a source of potential zoonoses, we collected throat and rectal swabs from 334 Sunda pangolins (*Manis javanica*) confiscated in Peninsular Malaysia and Sabah between August 2009 and March 2019. Total nucleic acid was extracted for viral molecular screening using conventional PCR protocols used to routinely identify known and novel viruses in extensive prior sampling (> 50,000 mammals). No sample yielded a positive PCR result for any of the targeted viral families—Coronaviridae, Filoviridae, Flaviviridae, Orthomyxoviridae and Paramyxoviridae. In the light of recent reports of coronaviruses including a SARS-CoV-2-related virus in Sunda pangolins in China, the lack of any coronavirus detection in our ‘upstream’ market chain samples suggests that these detections in ‘downstream’ animals more plausibly reflect exposure to infected humans, wildlife or other animals within the wildlife trade network. While confirmatory serologic studies are needed, it is likely that Sunda pangolins are incidental hosts of coronaviruses. Our findings further support the importance of ending the trade in wildlife globally.

## Introduction

The legal and illegal trade in wildlife for consumption as food, medicine and other products is a globally significant threat to conservation (Smith et al. 2006; Nayar 2009; Rosen and Smith 2010). It also drives the emergence of pathogens that threaten human and domestic animal health, and national and global economies (Lee and McKibbin 2004; Smith et al. 2008, 2009). This includes the 2003 Severe Acute Respiratory Syndrome (SARS) outbreak caused by SARS coronavirus (SARS-CoV), which originated in the large wet markets of Guangdong province, China (Ksiazek et al. 2003), and the current COVID-19 outbreak caused by SARS-CoV-2, first discovered in people associated with a wet market in Wuhan (Zhou et al. 2020; Zhu et al. 2020). Both viruses likely originated in bats, with SARS-CoV infecting civets and other small mammals in the markets, which may have acted as intermediate or amplifying hosts (Guan et al. 2003; Wang and Eaton 2007). The finding of furin cleavage insertions in the spike (s) protein sequence in the SARS-CoV-2 genome has led some to suggest that intermediate hosts may have been involved in the emergence of COVID-19 (Andersen et al. 2020); however, no intermediate hosts have so far been conclusively identified. Recently, four different groups have identified coronaviruses in imported Sunda or Malayan pangolins (*Manis javanica*) seized in raids on wildlife traders in China (Liu et al. 2019; Lam et al. 2020; Xiao et al. 2020; Zhang et al. 2020). The genomes of these are closely related to SARS-CoV-2, particularly in some genes, including the s-gene responsible for binding to host cells, albeit that some bat-CoVs have higher overall sequence identity to SARS-CoV-2 (Latinne et al. 2020). Authors of these papers propose that further sampling of pangolins might help elucidate the potential role of pangolins in the evolution of SARSr-CoVs, the emergence of COVID-19, and the risk of future zoonotic viral emergence (Liu et al. 2020; Lam et al. 2020; Xiao et al. 2020).

Over a ten-year period, as part of the USAID PREDICT project (PREDICT Consortium 2017; PREDICT Consortium 2019), we collected biological samples from confiscated and rescued Sunda pangolins in their country of origin: Peninsular Malaysia and the Malaysian state of Sabah on the island of Borneo. The aims of this study were to identify the phylogeographic origins of confiscated pangolins and any potentially zoonotic pathogens associated with them (Karesh 2010). Here, we report on the results from pathogen surveillance and discovery screening of these pangolin samples.

## Materials and Methods

**Figure 1:**
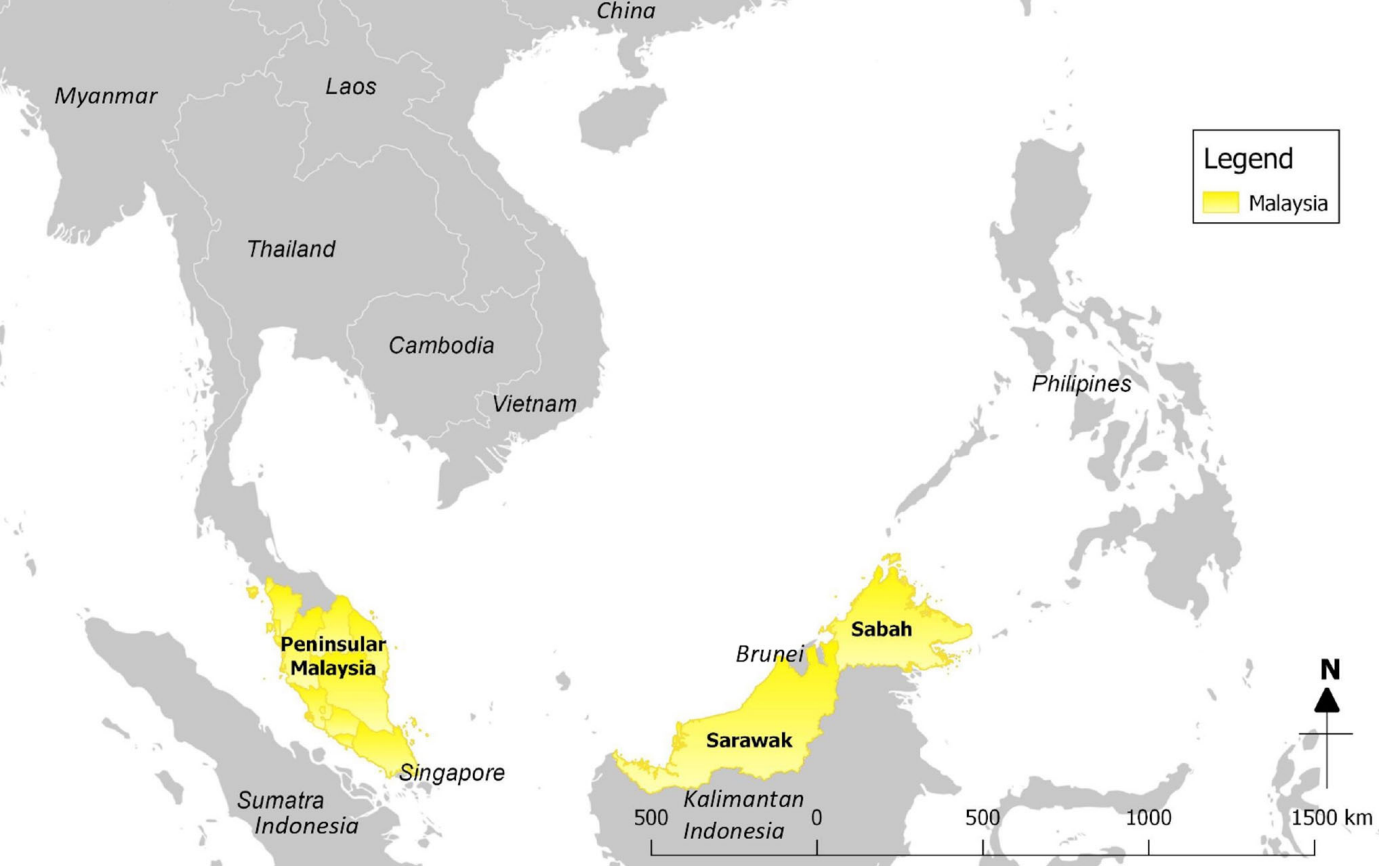
Map of Southeast Asia and China.

**Figure 2:**
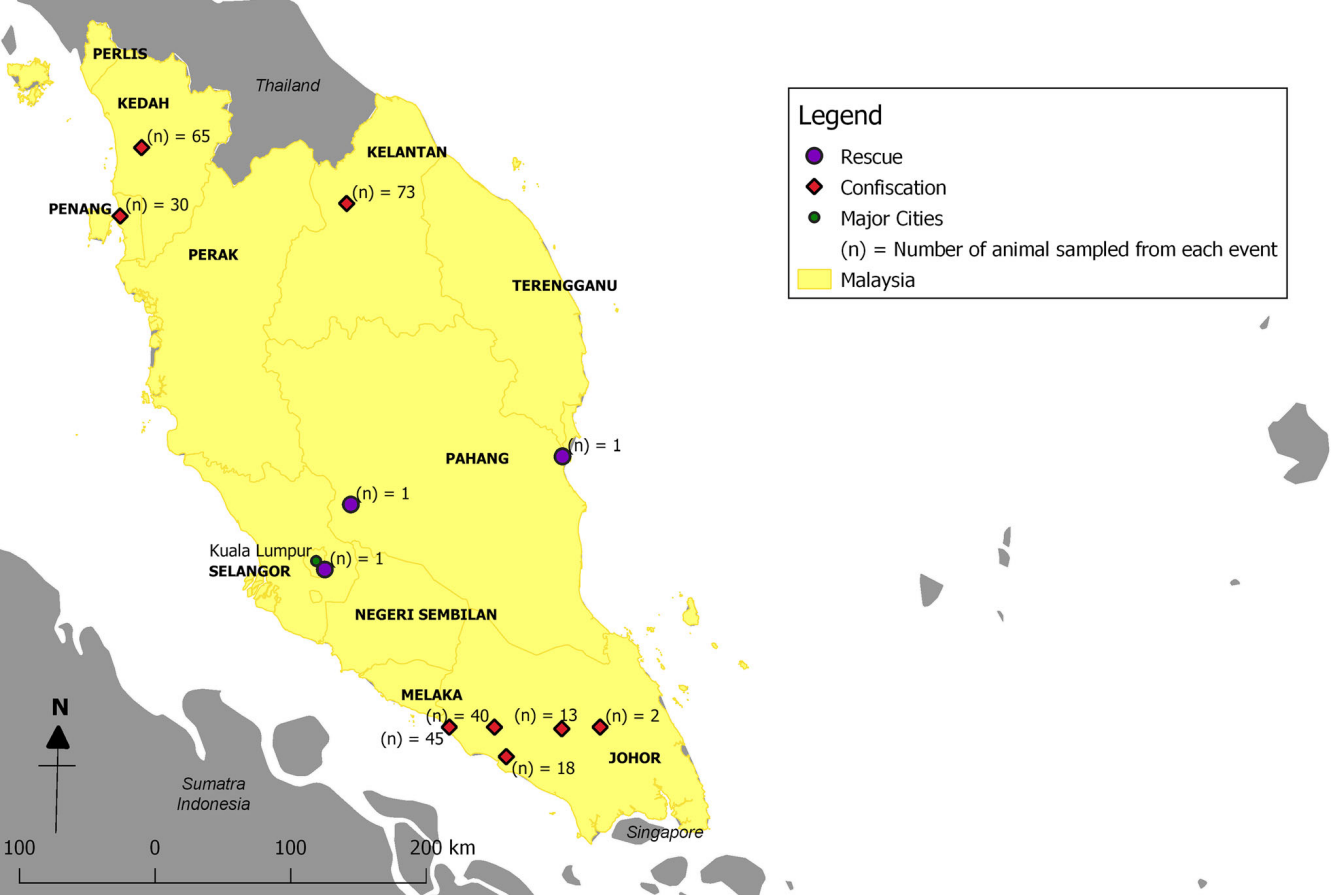
Locations where pangolins were rescued and confiscated in Peninsular Malaysia.

**Figure 3:**
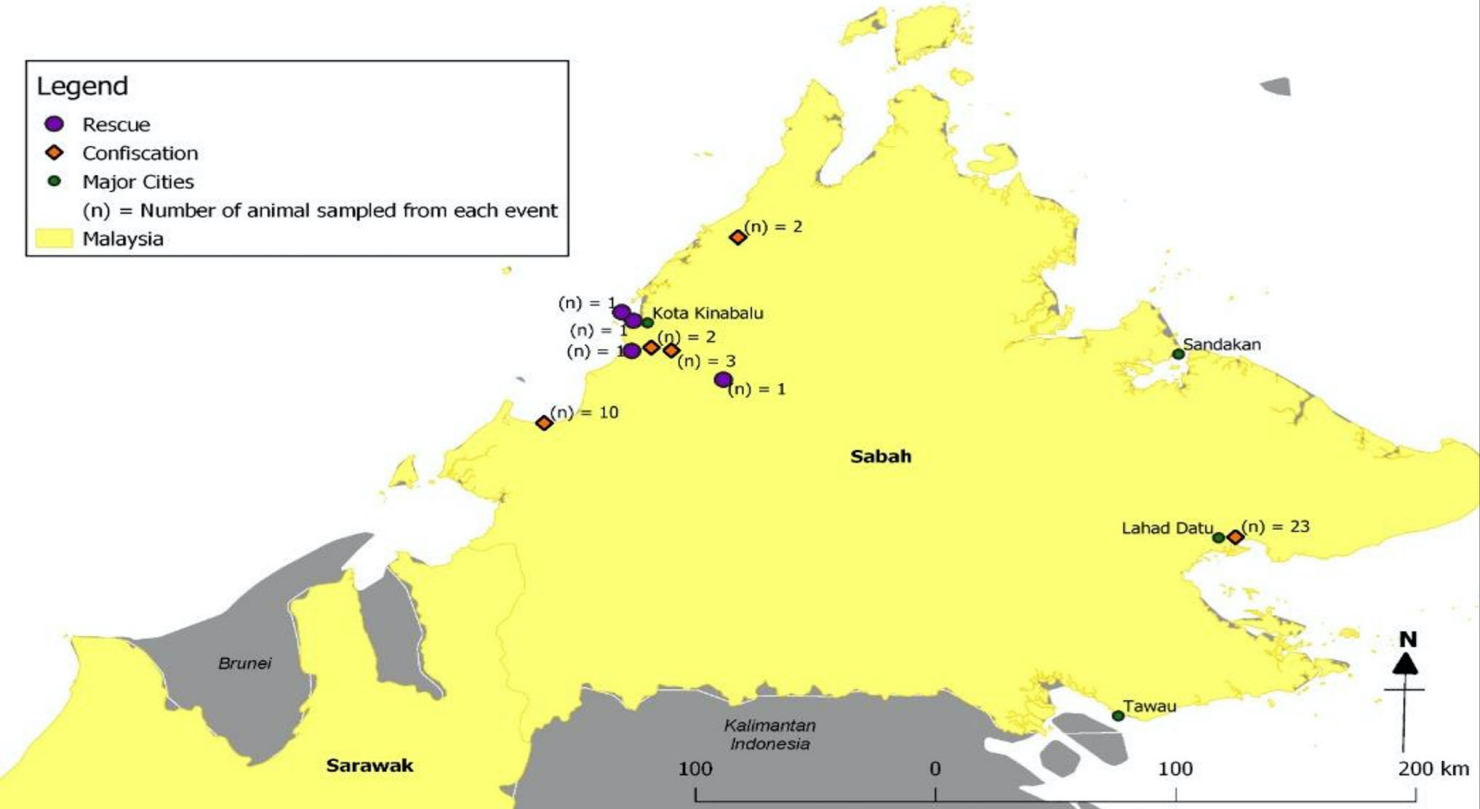
Locations where pangolins were rescued and confiscated in Sabah state.

Sunda pangolins (*Manis javanica*) were either confiscated from smugglers or rescued from the wild between August 2009 and March 2019 and were in the possession of the Department of Wildlife and National Parks Peninsular Malaysia, or Sabah Wildlife Department at the time of sampling or Sabah Wildlife Department at the time of sampling (Figs. 1, 2, 3). Most confiscations occurred near national borders or ports and were reported to be destined for other Southeast Asian countries en route to China and were usually found in sacks or crates in temporary holding facilities, or in vehicles. The wild-rescued Sunda pangolins were all surrendered by members of the public who found them in their native habitats. All pangolins were alive during the sampling process; based on their weakened condition, it appeared that some had been in captivity for many weeks, although we were unable to confirm this.

The sampling protocol was approved by UC DAVIS Institutional Animal Care and Use Committee (protocol number: 16048). Each pangolin was assigned a unique identification code; GPS coordinates of the confiscation or rescue locations, biometric measurement and physical health check information were recorded. Swab samples were collected from the throat and rectum using a sterile non-absorbent mini-tip polyester swab (Puritan, Guilford, USA) placed in a cryotube contained 600 μL of TRIzol reagent (Invitrogen, Carlsbad, USA) to maintain RNA integrity. All samples were stored at − 196°C immediately after collection (typically within 10 min) in a liquid nitrogen dewar MVE Doble 34 (Chart Biomed, Ball Ground, USA) in the field and transferred to a − 80°C freezer for long-term storage on return to the laboratory. Cold chain integrity in storage was ensured by carbon dioxide monitoring, temperature monitoring, and a backup generator system, consistent with certified BSL-2 laboratory requirements.

Total nucleic acid was extracted for viral molecular screening using the NUCLISENS EASYMAG or MINIMAG system according to the manufacturer’s protocol with validated modifications (bioMérieux, Marcy l’Etoile, France). The purity and concentration of extracted RNA were assessed using the Nanodrop 2000 spectrophotometer (Thermo Scientific, Massachusetts, USA). Complementary DNA (cDNA) of each sample was generated, according to manufacturer’s protocol with random hexamers, from the SuperScript III First-Strand Synthesis System for reverse transcription PCR (Invitrogen, Carlsbad, USA). cDNA synthesis control from the kit was performed as per manufacturer’s protocol using the control RNA and the control sense and control antisense primers. The synthesized cDNA was validated with PCR targeting the cytochrome c oxidase subunit I (CO1) gene and the cytochrome b (Cytb) mitochondrial gene (Table 1). The cDNA was used in conventional PCR protocols screening five viral families: Coronaviridae, Filoviridae, Flaviviridae, Orthomyxoviridae and Paramyxoviridae (Table 1). The PCRs were conducted in a Veriti or SimpliAmp thermal cycler (Applied Biosystems, Foster City, USA). Reactions were carried out in a final volume of 20 µl, following the manufacturer’s protocol (Qiagen, Hilden, Germany) using 1 µl of the cDNA product as a template and either Fast-Cycling PCR kit or HotStarTaq *Plus* Master Mix with a final concentration of 0.1 µM for each primer following the manufacturer’s protocols (Qiagen, Hilden, Germany). PREDICT universal controls 1 and 2 (Anthony et al. 2013) and specific controls for Filovirus (One Health Institute Laboratory, University of California, Davis) and Influenza Liang PCR (Liang et al., unpublished) were used. Peninsular Malaysia and Sabah samples were screened on separate occasions at two different certified BSL2 biocontainment level laboratories using standardised methods. PCR products were loaded and run on 1% agarose gel electrophoresis − 100 V, for 30–45 min with 0.5 × tris–acetate-EDTA buffer (Vivantis Technologies Sdn. Bhd., Subang Jaya, Malaysia). The gels were viewed on a transilluminator, and expected size bands were excised and stored in separate microcentrifuge tubes, and the corresponding post-PCR mixes were used as a template for contamination control PCRs to check for contamination from the universal positive controls. PCR products were run under the same gel electrophoresis conditions; those without the expected size bands showed that there was no contamination from the controls. Products from the initial PCR of these samples were then purified using the Ultrafree-DA centrifugal filter units (Millipore, Cork, Ireland); the purified products were cloned using the dual-colour selection Strataclone PCR cloning kit according to the manufacturer’s protocol (Stratagene, La Jolla, USA). Up to eight colonies containing the PCR product were selected and inoculated on Luria–Bertani agar slants, individually. Grown colonies were sent to a commercial company for direct colony sequencing.

**Table 1:** PCR Conditions and Primer Sequences Used.

Target gene and product length	Primers	Conditions	Reference
Cytochrome c oxidase subunit I gene 750 bp	BatL5310 F: CCTACTCRGCCATTTTACCTATG R6036R R: ACTTCTGGGTGTCCAAAGAATCA	94°C for 5 min, then 35 cycles of 94°C for 30 s, 48°C for 45 s and 72°C for 90 s. Finish with 72°C for 10 min	Robins et al. (2007)
Cytochrome b mitochondrial gene ~457 bp	Cytb F: GAGGMCAAATATCATTCTGAGG Cytb R: TAGGGCVAGGACTCCTCCTAGT	95°C for 2 min, then 50 cycles of 94°C for 30 s, 52°C for 50 s and 71°C for 1 min. Finish with 72°C for 7 min	Townzen et al. (2008)
Coronaviridae RNA-Dependent RNA Polymerase (RdRp) First Round: 520 bp Second Round: 328 bp	Round 1: CoV-FWD1: CGTTGGIACWAAYBTVCCWYTICARBTRGG CoV-RVS1: GGTCATKATAGCRTCAVMASWWGCNACATG Round 2: CoV-FWD2: GGCWCCWCCHGGNGARCAATT CoV-RVS2: GGWAWCCCCAYTGYTGWAYRTC	95°C for 5 min, then 40 cycles of 96°C for 5 s, 60°C for 8 s and 68°C for 15 s. Finish with 72°C for 2 min Same protocol for rounds 2 but for 35 cycles. Designed to be used with Fast-Cycling PCR kit	Quan et al. (2010)
Coronaviridae RdRp First Round: 440 bp Second Round: 434 bp	Round 1: CoV-FWD3: GGTTGGGAYTAYCCHAARTGTGA CoV-RVS3: CCATCATCASWYRAATCATCATA Round 2: *CoV*-*FWD4/Other:* GAYTAYCCHAARTGTGAUMGWGC CoV-RVS3: CCATCATCASWYRAATCATCATA	95°C for 5 min, then 40 cycles of 96°C for 5 s, 60°C for 8 s and 68°C for 12 s. Finish with 72°C for 2 min Same protocol for rounds 1 and 2. Designed to be used with Fast-Cycling PCR kit	Modified from Watanabe et al. (2010)
Filoviridae L-Gene First Round: 680 bp Second Round: 630 bp	Round 1: Filo-MOD-FWD: TITTYTCHVTICAAAAICAYTGGG FiloL.conR: ACCATCATRTTRCTIGGRAAKGCTTT Round 2: Filo-MOD-FWD: TITTYTCHVTICAAAAICAYTGGG Filo-MOD-RVS: GCYTCISMIAIIGTTTGIACATT	94°C for 5 min, followed by 40 cycles of 94°C for 1 min, 52°C for 1 min and 72°C for 1 min. Finish with a final extension of 72°C for 7 min Same protocol for rounds 1 and 2	Zhai et al. (2007)
Flaviviridae NS5 gene ~270 bp	Flavi-FWD: TGYRTBTAYAACATGATGGG Flavi-RVS: GTGTCCCAICCNGCNGTRTC	95°C for 5 min followed by 45 cycles of 94°C for 15 s, 50°C for 30 s, 72°C for 45 s, and 77°C for 15 s. Finish with 72°C for 10 min	Moureau et al. (2007)
Orthomyxoviridae M gene 243 bp	FLUAV-M-U44: GTCTTCTAACCGAGGTCGAAACG FLUAV-M-L287: GCATTTTGGACAAAGCGTCTACG	94°C for 2 min, then 45 cycles of 94°C for 30 s, 52°C for 30 s and 72°C for 30 s. Finish with 72°C for 7 min	Anthony et al. (2012)
Orthomyxoviridae PB1 gene First Round: 407 bp Second Round: 402 bp	Round 1 FLUAPB1-F: ATGATGATGGGNATGTTYAAYATG FLUAPB1-R: GCNGGNCCNAKDTCRYTRTTDATCAT Round 2 FLUAPB1-NF: GATGGGNATGTTYAAYATGYTDAGYAC FLUAPB1-R: Same reverse primer as Round 1	95°C for 5 min, then 14 cycles of 95°C for 30 s, 65°C for 35 s (-1°C/cycle) and 72°C for 50 s. Then perform 35 cycles of 95°C for 30 s, 50°C for 30 s and 72°C for 50 s. Finish with 72°C for 7 min Same protocol for rounds 1 and 2	Liang, unpublished. Developed at Center for Infection and Immunity
Paramyxoviridae Polymerase (*pol*) gene First round: ~ 639 bp Second round: ~561 bp	Round 1: PAR-F1: GAAGGITATTGTCAIAARNTNTGGAC PAR-R: GCTGAAGTTACIGGITCICCDATRTTNC Round 2: PAR-F2: GTTGCTTCAATGGTTCARGGNGAYAA PAR-R: GCTGAAGTTACIGGITCICCDATRTTNC	Round 1: 94°C for 5 min, followed by 40 cycles of 94°C for 1 min, 48°C for 1 min and 72°C for 1 min. Finish with a final extension of 72°C for 7 min Round 2: 95°C for 5 min, followed by 40 cycles of 96°C for 5 s, 48°C for 8 s and 68°C for 15 s. Finish with 72°C for 3 min. Designed to be used with Fast-Cycling PCR kit	Tong et al. (2008)
Contamination control PCR for Universal Control 1 412 bp	PREDICT-Fwd: GGGCCTAGAGAAGATATTTGTACT PREDICT-Rvs: CGCC ATTGACATCCTCGAAG	94°C for 2 min, then 40 cycles of 94°C for 30 s, 55°C for 30 s, 72°C for 1 min. Finish with 72°C for 2 min	Unpublished. Designed at CII
Contamination control PCR for Universal Control 2 318 bp	DAVIS-Fwd: CGACTCACTATAGGGAGAGACTTCG DAVIS-Rvs: CCGAGTTACATAACGCTTTGATTGCC	94°C for 2 min, then 40 cycles of 94°C for 30 s, 54°C for 30 s, 72°C for 1 min. Finish with 72°C for 2 min	Unpublished. Designed at University of California, Davis

## Results

**Figure 4:**
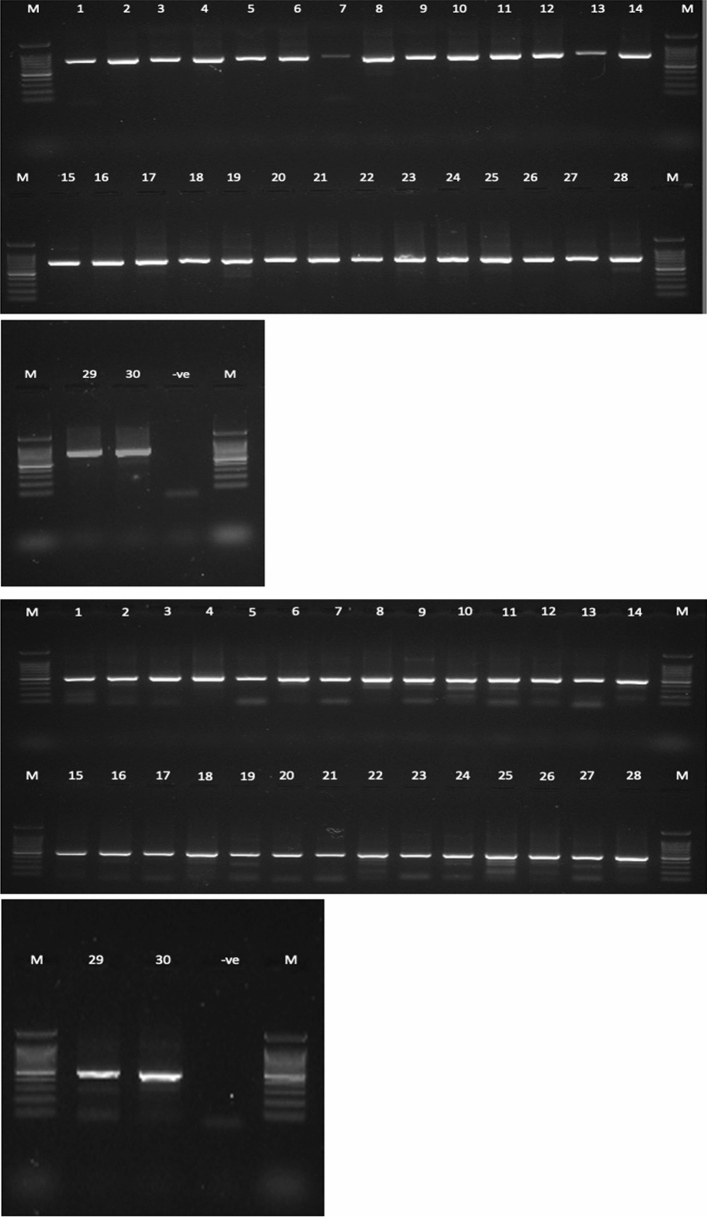
**a** 1% agarose gel electrophoresis of PCR targeting on cytochrome c oxidase subunit I gene for a subset of pangolin samples. [Lane M: 100 bp DNA ladder; Lane 1—ZEN00950T, Lane 2—ZEN00950R, Lane 3—ZEN00951T, Lane 4—ZEN00951R, Lane 5—ZEN00952T, Lane 6—ZEN00952R, Lane 7—ZEN00953T, Lane 8—ZEN00953R, Lane 9—ZEN00954T, Lane 10—ZEN00954R, Lane 11—ZEN00955T, Lane 12—ZEN00955R, Lane 13—ZEN00956T, Lane 14—ZEN00956R, Lane 15—ZEN00957T, Lane 16—ZEN00957R, Lane 17—ZEN00958T, Lane 18—ZEN00958R, Lane 19—ZEN00959T, Lane 20—ZEN00959R, Lane 21—ZEN00960T, Lane 22—ZEN00960R, Lane 23—ZEN00961T, Lane 24—ZEN00961R, Lane 25—ZEN00962T, Lane 26—ZEN00962R, Lane 27—ZEN00963T, Lane 28—ZEN00963R, Lane 29—ZEN00964T, Lane 30—ZEN00964R, Lane (-ve): Negative control].** b** 1% agarose gel electrophoresis of PCR targeting on cytochrome b gene for a subset of pangolin samples. [Lane M: 100 bp DNA ladder; Lane 1—ZEN00950T, Lane 2—ZEN00950R, Lane 3—ZEN00951T, Lane 4—ZEN00951R, Lane 5—ZEN00952T, Lane 6—ZEN00952R, Lane 7—ZEN00953T, Lane 8—ZEN00953R, Lane 9—ZEN00954T, Lane 10—ZEN00954R, Lane 11—ZEN00955T, Lane 12—ZEN00955R, Lane 13—ZEN00956T, Lane 14—ZEN00956R, Lane 15—ZEN00957T, Lane 16—ZEN00957R, Lane 17—ZEN00958T, Lane 18– ZEN00958R, Lane 19—ZEN00959T, Lane 20—ZEN00959R, Lane 21—ZEN00960T, Lane 22—ZEN00960R, Lane 23—ZEN00961T, Lane 24—ZEN00961R, Lane 25—ZEN00962T, Lane 26—ZEN00962R, Lane 27—ZEN00963T, Lane 28—ZEN00963R, Lane 29—ZEN00964T, Lane 30—ZEN00964R, Lane (-ve): Negative control].

A total of 334 Sunda pangolins were screened: 289 in Peninsular Malaysia (confiscated *n* = 286; wild-rescued *n* = 3) (Table 2a, b) and 45 in Sabah state (confiscated *n* = 40; wild-rescued *n* = 5) (Table 2c, d). No sample yielded a positive PCR result for any member of the targeted virus families, either in Peninsular Malaysia (95% CI 0.0–0.01) or in Sabah (95% CI 0.0–0.08). RNA extracted from the samples had low purity and concentration (Table 3); however, both the CO1 gene (Fig. 4a) and Cytb gene (Fig. 4b) were successfully amplified by PCR. All positive controls were successfully amplified, confirming that the PCRs were performing properly.

**Table 2:** Details of Sunda pangolins.

No.	Location	Date	Interface
*(a) Rescued from the wild in Peninsular Malaysia*			
1	(**) Kuantan, Pahang (*n* = 1)	11/07/2018	Rescued from wild
2	(**) Bentong, Pahang (*n* = 1)	29/09/2018	Rescued from wild
3	(**) Kuantan, Pahang (*n* = 1)	25/03/2019	Rescued from wild
*(b) Confiscated from Peninsular Malaysia*			
1	(*) Kedah (*n* = 65)	27/08/2009	Confiscation
2	(*) Kelantan (*n* = 73)	09/09/2009	Confiscation
3	(*) Johor (*n* = 18)	30/09/2009	Confiscation
4	(*) Johor (*n* = 2)	06/11/2009	Confiscation
5	(*) Johor (*n* = 40)	07/11/2009	Confiscation
6	(*) Johor (*n* = 45)	06/04/2010	Confiscation
7	(*) Pulau Pinang (*n* = 30)	27/04/2010	Confiscation
8	(*) Johor (*n* = 13)	22/07/2010	Confiscation
*(c) Rescued from the wild in Sabah state*			
1	(**) Tambunan (*n* = 1)	17/06/2015	Rescued from wild
2	(**) Kota Kinabalu (*n* = 1)	01/05/2016	Rescued from wild
3	(**) Penampang (*n* = 1)	07/06/2016	Rescued from wild
4	(**) Kota Kinabalu (*n* = 1)	21/02/2017	Rescued from wild
5	(**) Penampang (*n* = 1)	18/01/2018	Rescued from wild
*(d) Confiscated from Sabah state*			
1	(**) Beaufort (*n* = 10)	31/10/2014	Confiscation
2	(**) Kota Belud (*n* = 2)	18/09/2015	Confiscation
3	(**) Lahad Datu (*n* = 23)	22/02/2016	Confiscation
4	(**) Penampang (*n* = 2)	29/11/2016	Confiscation
5	(**) Sandakan (*n* = 3)	26/09/2017	Confiscation

(a), (b), (c) and (d) Details of Sunda pangolins confiscated from smugglers and rescued from wild. (Level of detail for the location of confiscations and rescue events reported was determined by the local wildlife departments).

(*n*) indicates the total number of pangolins sampled in the event.

(*) indicates the state where the confiscation or rescue occurred in Peninsular Malaysia.

(**) indicates the district where the confiscation or rescue occurred in Peninsular Malaysia or Sabah state.

**Table 3:** RNA Purity and Concentration for a Subset of Pangolin Samples Accessed by Using Nanodrop Spectrophotometer.

No.	Sample ID	Nucleic acid (ng/µL)	A260 (nm)	A280 (nm)	260/280 (nm)	Factor
1	ZEN00950T	1.1	0.027	0.036	0.13	40
2	ZEN00950R	6.7	0.168	0.148	2.07	40
3	ZEN00951T	4.3	0.108	0.115	1.11	40
4	ZEN00951R	19.7	0.493	0.365	1.16	40
5	ZEN00952T	3.6	0.091	0.092	1.08	40
6	ZEN00952R	20.0	0.500	0.343	3.08	40
7	ZEN00953T	5.8	0.146	0.159	1.12	40
8	ZEN00953R	11.5	0.287	0.194	2.68	40
9	ZEN00954T	3.2	0.079	0.096	0.33	40
10	ZEN00954R	3.6	0.090	0.088	0.34	40
11	ZEN00955T	5.3	0.133	0.118	0.52	40
12	ZEN00955R	36.1	0.902	0.573	1.01	40
13	ZEN00956T	9.2	0.230	0.25	0.52	40
14	ZEN00956R	6.2	0.154	0.116	1.21	40
15	ZEN00957T	0.9	0.024	0.042	0.07	40
16	ZEN00957R	13.9	0.347	0.255	4.21	40
17	ZEN00958T	9.3	0.232	0.213	1.28	40
18	ZEN00958R	11.5	0.287	0.184	2.18	40
19	ZEN00959T	5.2	0.130	0.162	1.63	40
20	ZEN00959R	11.8	0.296	0.321	2.26	40
21	ZEN00960T	8.8	0.220	0.241	1.00	40
22	ZEN00960R	15.1	0.377	0.324	2.16	40
23	ZEN00961T	12.1	0.303	0.356	1.54	40
24	ZEN00961R	13.0	0.728	0.532	1.23	40
25	ZEN00962T	29.1	0.325	0.234	0.88	40
26	ZEN00962R	17.0	0.426	0.319	0.88	40
27	ZEN00963T	13.2	0.331	0.317	0.79	40
28	ZEN00963R	6.6	0.166	0.115	1.16	40
29	ZEN00964T	1.7	0.044	0.074	0.11	40
30	ZEN00964R	6.5	0.162	0.143	0.56	40

## Discussion

Our negative findings across five viral families associated with emerging and re-emerging zoonotic diseases in recent decades in these throat and rectal swabs indicate the absence of viral shedding, and contrast with reports of the detection of parainfluenza virus (Wang et al. 2018), coronaviruses and Sendai virus (Liu et al. 2019; Zhang et al. 2020), and SARSr-CoVs (Lam et al. 2020; Xiao et al. 2020) in Sunda pangolins. Our sample size is substantial, particularly given the rarity of these animals in Malaysia—the International Union for Conservation of Nature (IUCN) lists the Sunda pangolin (*Manis javanica*) as ‘Critically Endangered’, as a result of poaching, smuggling and habitat loss (IUCN 2018). Our previous studies of bat coronaviruses revealed 5–10% PCR prevalence (Yang et al. 2016; Anthony et al. 2017; Hu et al. 2017; Latinne et al. 2020), suggesting that even at the upper limit of the 95% confidence interval, our negative findings in pangolins are inconsistent with coronavirus shedding and endemic coronavirus infection at a population level. Serologic studies are needed to support this contention. Although the RNA had low purity and concentration (Table 3), we repeatedly detected coronaviruses using the same protocols in bat samples that had similar RNA purity and concentration and had been stored for > 5 years (Table 4) as part of the PREDICT project (PREDICT Consortium 2017; PREDICT Consortium 2019). We are confident that the PCR protocols were sensitive and robust and that if coronaviruses had been present in these samples at the same viral load, they would have detected them.

**Table 4:** RNA Purity and Concentration for a Subset of Bat Samples (Tested PCR Positive) Accessed by Using Nanodrop Spectrophotometer.

No.	Sample ID	Nucleic acid (ng/µL)	A260 (nm)	A280 (nm)	260/280 (nm)	Factor
1	PMW01285T	15.1	0.377	0.274	1.38	40
2	PMW01285R	5.7	0.143	0.132	1.08	40
3	PMW01288T	2.5	0.064	0.063	1.00	40
4	PMW01288R	26.8	0.67	0.496	1.35	40
5	PMW01455T	10.0	0.249	0.269	0.93	40
6	PMW01455R	5.7	0.143	0.135	1.05	40
7	PMW01607T	6.2	0.155	0.193	0.8	40
8	PMW01607R	4.7	0.118	0.169	0.7	40
9	PMW01935T	8.5	0.213	0.234	0.91	40
10	PMW01935R	9.2	0.230	0.292	0.79	40

While our sampling was necessarily opportunistic (given the conservation status and the cryptic nature of the species) and sampling intensity varied, our negative findings over ten years and at multiple locations support the veracity of the findings. The most parsimonious explanation for the contrast between our findings and the discovery of SARSr-CoVs in Sunda pangolins by (Liu et al. 2019, 2020; Lam et al. 2020; Xiao et al. 2020; Zhang et al. 2020) is the nature of the sampled population: our samples were drawn from an ‘upstream’ cohort of animals yet to enter or just entering the illegal trade network, whereas all others were drawn from ‘downstream’ cohorts confiscated at their destination in China. During the wildlife trade transits, which often includes movement through other Southeast Asian countries, animals are often housed together in groups from disparate geographic regions, and often with other species, giving opportunity for viral transmission among and within species. This is supported by data from bamboo rats in the wildlife market chain in Vietnam that had increasing prevalence of coronaviruses as they moved from traders, to large markets, to restaurants, concomitant with length of time in the wildlife trade (Huong et al. 2020). The housing of some of the animals in rehabilitation centres in China would also allow for exposure to coronaviruses from other groups or species. In natural wildlife reservoir hosts, SARSr-CoVs appear to cause little if any clinical signs, and this is supported by the limited laboratory infections so far carried out (Watanabe et al. 2010). The reports of clinical illness and pathology associated with coronavirus infection in pangolins (Liu et al. 2019; Xiao et al. 2020) are unlikely in a reservoir host. We therefore conclude that the detections of SARS-CoV-2-related viruses in pangolins are more plausibly a result of their exposure to infected people, wildlife or other animals after they entered the trade network. Thus, the likelihood is that Sunda pangolins are incidental rather than reservoir hosts of coronaviruses as claimed by Zhang et al. (2020).

Our microsatellite DNA fragment analysis (manuscript in preparation) suggests that confiscated pangolins from Peninsular Malaysia and Sabah were taken from Malaysia, Brunei or Indonesia; however, further analysis of pangolins from the neighbouring countries is required to confirm the results. They were confiscated at holding facilities, ports or borders prior to shipment, and had not yet been exposed to multiple potential sources of infection, unlike the confiscated animals in China reported by Xiao et al. (2020) and Lam et al. (2020). An array of pathogens and infections have been observed in wet markets, in wildlife (Dong et al. 2007; Cantlay et al. 2017), in humans (Xu et al. 2004) and in domestic animals (Karesh et al. 2005). In comparison with wildlife screened from the wild (Poon et al. 2004) and from farms (Tu et al. 2004; Kan et al. 2005), wildlife in markets have a much higher chance of exposure to pathogens and disease spillover. These findings highlight the importance of carefully and systemically ending the trade in wildlife and improving biosecurity to avoid having wet markets where wild animals are mixing with farmed animals and humans.

Our findings suggest that pangolins that have not entered the illegal wildlife trade pose no threat to human health. While the detection of SARS-CoV-2 like viruses in some trade-rescued pangolins suggests a parallel with traded civets (*Parguma larvata*) in the emergence of SARS-CoV (Guan et al. 2003), any role as an intermediate host in the transmission of SARS-CoV-2 from a putative natural bat host to humans is yet to be established. It is also important to note that phylogenetic comparison of the whole genomes and the RdRp sequence of the SARSr-CoVs sequenced from pangolins suggests that these are recombinants between a bat and a pangolin CoV and that there are two other bat-CoVs that are more closely related to SARS-CoV-2 (Latinne et al. 2020). Serological studies in pre-trade pangolins will shed further light on any role of pangolins as hosts of SARS-CoV-2-related viruses. All pangolin species face known and significant threats to their survival in nature and require active conservation efforts to ensure their enduring existence for future generations.
